# The Effects of Quercetin on Vascular Endothelium, Inflammation, Cardiovascular Disease and Lipid Metabolism—A Review

**DOI:** 10.3390/nu17091579

**Published:** 2025-05-03

**Authors:** Mateusz Ozorowski, Michał Wiciński, Oskar Kuźmiński, Paweł Wojciechowski, Zygmunt Siedlecki, Maciej Śniegocki, Elżbieta Włodarczyk

**Affiliations:** 1 Department of Pharmacology and Therapeutics, Faculty of Medicine, Collegium Medicum in Bydgoszcz, Nicolaus Copernicus University, M. Curie 9, 85-090 Bydgoszcz, Poland; 2 Department of Neurosurgery and Neurotraumatology and Pediatric Neurosurgery, Collegium Medicum in Bydgoszcz, Nicolaus Copernicus University, M. Curie 9, 85-090 Bydgoszcz, Poland; 3 Department of Geriatrics, Faculty of Medical Sciences, Collegium Medicum in Bydgoszcz, Nicolaus University, M. Curie 9, 85-090 Bydgoszcz, Poland

**Keywords:** quercetin, inflammation, cardiovascular diseases, vascular endothelium, lipid metabolism disorder

## Abstract

Quercetin is a naturally occurring flavonoid of plant origin. This naturally occurring polyphenolic compound is generally known for its antioxidant and anti-inflammatory properties and has been reported to be a factor in improving the antioxidant defense system, lipid metabolism, and reducing the incidence of cardiovascular and inflammatory diseases. In this article, we will take a closer look at quercetin—what it is, what properties it has, what health benefits it can bring, and in which products it can be found. Thanks to its wide spectrum of action, quercetin is gaining popularity as an ingredient in dietary supplements, as well as an element of a healthy diet supporting overall health.

## 1. Introduction

Quercetin, a powerful antioxidant and anti-inflammatory compound from the polyphenol group, may play a significant role in modulating inflammatory processes and oxidative stress, which underlie many chronic diseases, such as cardiovascular and lipids metabolism disorders. Numerous studies confirm its effect on modulating cellular and molecular processes associated with inflammatory responses and protection against oxidative damage. Atherosclerosis remains a leading cause of morbidity and mortality worldwide, underlying conditions such as coronary artery disease, stroke, and peripheral artery disease. It is characterized by the accumulation of lipids, inflammatory cells, and fibrous elements in the arterial walls, leading to the formation of atheromatous plaques [1,2]. These plaques can compromise arterial integrity and function, resulting in reduced blood flow and potential plaque rupture, which can precipitate acute cardiovascular events. In recent years, there has been increasing interest in dietary quercetin as a therapeutic agent in the prevention and management of atherosclerosis [1,2,3].

**Figure 1:**
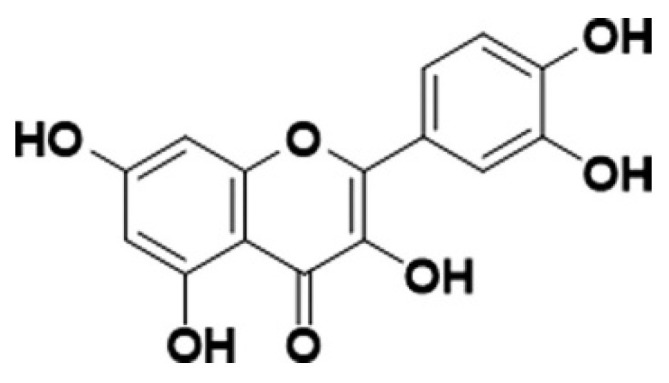
Chemical structure of quercetin [6].

**Figure 2:**
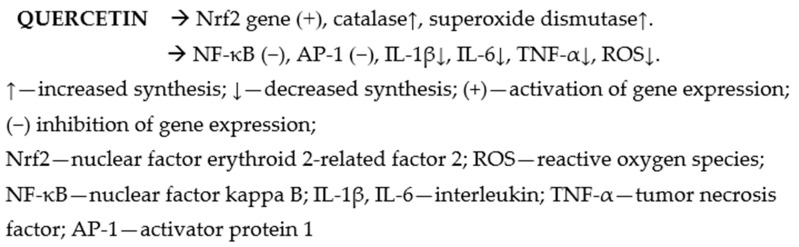
The effect of quercetin on inflammation and ROS [11,15,16].

Quercetin is commonly found in a variety of fruits and vegetables, including apples, berries, mango fruit, asparagus, onions, Malanbar tamarind, and St. John’s wort (Hypericum perforatum). This flavonoid has received particular attention for its powerful antioxidant, anti-inflammatory, and vascular-protective properties [4,5]. Quercetin (3,3′,4′,5,7-pentahydroxyflavone) (Figure 1) is a naturally occurring polyphenolic flavonoid widely distributed in plants, including green tea [6,7]. The interest in quercetin stems from its diverse biological activities and potential therapeutic applications [8,9]. It exhibits significant anti-inflammatory effects by modulating key signaling pathways and inhibits the activity of enzymes like cyclooxygenase (COX) and lipoxygenase (LOX), reducing the synthesis of pro-inflammatory mediators [10]. Additionally, quercetin suppresses the nuclear factor-kappa B (NF-κB) pathway (Figure 2), which plays a crucial role in inflammation. This compound is renowned for its potent antioxidant properties. It scavenges free radicals and chelates metal ions, thus protecting cells from oxidative stress. The antioxidant effect is attributed to its ability to donate electrons and stabilize reactive oxygen species (ROS) [11]. Quercetin induces apoptosis, inhibits cell proliferation, and disrupts the cell cycle in cancer cells. Mechanistically, it modulates multiple signaling pathways, including the p53, PI3K/Akt, and MAPK pathways [11,12]. It also exhibits anti-angiogenic properties, inhibiting the growth of new blood vessels essential for tumor growth [12].

The molecular mechanisms underlying the beneficial effects of quercetin on the cardiovascular system are complex and multifaceted, involving the modulation of oxidative stress, inhibition of inflammatory pathways, improvement of endothelial function, and reduction in lipid accumulation [13]. The cardioprotective effects of quercetin are attributed to its antioxidant and anti-inflammatory properties. By improving endothelial function, it reduces blood pressure and inhibits the oxidation of low-density lipoprotein (LDL) cholesterol [14]. These effects collectively contribute to a reduced risk of cardiovascular diseases [13,14].

## 2. Vascular Endothelium

Due to potential anti-inflammatory and antioxidant properties, quercetin may have an influence on vascular endothelial cells, which may contribute to improving blood vessel function and limit the development of atherosclerosis and other cardiovascular diseases.

It blocks the activation of the transcription factor NF-κB and the AP-1 signaling pathway, which are key in inducing inflammation in endothelial cells. It also reduces the level of pro-inflammatory cytokines, such as TNF-α, IL-1β, and IL-6, which are responsible for the severity of inflammation (Figure 2) [15,16]. Activation of the Nrf2 gene, responsible for the expression of antioxidant enzymes such as catalase and superoxide dismutase, as well as inhibition of the enzymes such as NADPH oxidase (NOX2), reduces the production of reactive oxygen species [17,18,19], while inhibition of caveolin-1 phosphorylation reduces endothelial permeability [20,21,22,23,24]. Quercetin stimulates eNOS phosphorylation and increases Ca^2+^ levels, which leads to increased NO bioavailability and improved endothelial function and enhanced mitochondrial function [25,26,27]. It can effectively regulate vascular function by modulating Eph/Cav-1 signaling and decreasing the expression of EphB4, EphrinB2, and *p*-Cav-1 [28]. It may increase the activity of CD39 enzyme, thus inhibiting platelet aggregation [28,29,30,31]. Some studies suggested that quercetin reduces the expression of adhesion molecules (ICAM-1, VCAM-1, and E-selectin), which are responsible for the attachment of leukocytes to the endothelium and their migration into tissues, thus limiting inflammatory processes and the development of atherosclerosis [32,33]. In one study, quercetin treatment exerted endothelial protective effects by inhibiting protein kinase and resulting in mitochondrial fragmentation [33].

## 3. Inflammation

Quercetin, a powerful antioxidant and anti-inflammatory compound, may play a significant role in modulating inflammatory processes and oxidative stress, which underlie many chronic diseases, such as respiratory, cardiovascular, neurodegenerative, and autoimmune diseases. Numerous studies confirm its effect on modulating cellular and molecular processes associated with inflammatory responses and protection against oxidative damage. One of the important mechanisms of quercetin’s action at the molecular level is the reduction in pro-inflammatory cytokine activity, such as IL-6, Il-17, TNF-α, and IL-1β, by blocking NF-κB and AMPK signaling pathways. It also inhibits NF-κB translocation to the cell nucleus (which limits the production of pro-inflammatory mediators) and IκBα phosphorylation, limiting the expression of genes responsible for inflammation, and inhibits the activation of AP-1 signaling pathways [34,35,36,37,38,39,40,41,42,43]. The regulation of NF-κB, AP-1, and AMPK/SIRT1/NF-κB signaling pathways additionally limits the expression of pro-inflammatory cytokines and the activity of oxidative enzymes. It also increases Nrf2 activity, enhancing antioxidant defense [44]. Pro-inflammatory and antioxidant enzymes are another target of quercetin. This flavonoid inhibits the activity of cyclooxygenase (COX-2) and lipoxygenase, which limits the synthesis of inflammatory mediators such as prostaglandins and leukotrienes. In addition, it reduces the activity of inducible nitric oxide synthase (iNOS), which leads to reduced levels of nitric oxide (NO) and nitrosative stress. This is very important in the inflammatory process. It improves the activity of antioxidant enzymes such as superoxide dismutase (SOD), catalase, and glutathione peroxidase, which are responsible for neutralizing ROS. It also increases the level of glutathione (GSH) [45,46,47]. Quercetin stabilizes the membranes of mast cells and basophils, preventing the release of histamine and pro-inflammatory mediators, i.e., mRNA Il-1b, Il-6, and Il-8. It causes a decrease in macrophage polarization and inhibits their pro-inflammatory profile M1 [45,48]. It also promotes autophagy and reduces the expression of adhesion molecules (ICAM-1, VCAM-1), which limits the migration of leukocytes to the site of inflammation, reducing inflammation. In autoimmune diseases, i.e., atopic dermatitis, it shows a therapeutic effect by reducing the levels of cytokines and inflammatory markers and IgE. It also inhibits the inflammatory response in lung diseases, reducing the production of inflammatory mediators and the activity of the NLRP3 inflammasome [49,50,51]. Quercetin also stimulates mitochondrial biogenesis and prevents mitochondrial damage, thus supporting the tissue regeneration process [52]. Quercetin reduces the level of hydroxyl radicals, hydrogen peroxide, superoxide anion, and nitric oxide, which are key to the development of oxidative stress. It effectively neutralizes ROS by inhibiting the expression of NADP oxidase (NOX2), reducing lipid peroxidation and protecting DNA and proteins from oxidative damage [53]. By regulating lipopolysaccharide metabolism and reducing the activity of lipid peroxidation products (MDA and 4-HNE), quercetin blocks changes in mitochondria stimulated by LPS and improves LPS homeostasis by activating VDR. Several studies have shown that quercetin reduces oxidative stress levels in models of cardiovascular, lung, neurodegenerative, and metabolic diseases. By activating the AMPK/SIRT1/NF-κB pathway, quercetin regulates the expression of genes encoding antioxidant enzymes, which strengthens the body’s defense mechanisms [23,54]. As is known, inflammation and oxidative stress play a significant role in the development of cardiovascular diseases. Proof of the role of the inflammatory process in the occurrence of cardiovascular diseases has led to special attention being paid to this process. Table 1 presents studies on the effect of quercetin on the vascular endothelium and the inflammatory process.

## 4. Cardiovascular Diseases

Cardiovascular diseases are a huge civilization challenge for modern medicine. In epidemiological data on European countries, deaths from cardiovascular causes are the most common cause in both women and men [57]. Classic cardiovascular risk factors include dyslipidemia, diabetes, hypertension, obesity, smoking, age, and genetic predisposition. The latest data indicate that extremely high air temperatures contribute to the increase in the number of deaths from cardiovascular causes [58]. It is therefore particularly important due to climate change and the occurrence of extreme weather conditions to lead to a reduction in cardiovascular risk. The introduction of drugs such as statins, ACEi, and flozin was a boon in reducing the risk [59]. The multifaceted nature of cardiovascular risk has led to changes in the latest guidelines for the treatment of chronic diseases, where the focus has begun on risk reduction, and the use of drugs with proven beneficial cardiovascular effects has been recommended. Researchers are also paying attention to new biological adaptogens that may be of potential benefit to the patient. These are safe compounds, occurring every day in our diet. Quercetin has proven to be an effective and safe therapeutic compound in relation to classical factors through its hypolipidemic, hypotensive, and hypoglycemic effects and suppression of cardiotoxicity; moreover, it has antioxidant, immunomodulatory, antibacterial, antiplatelet, and protective effects on the vascular endothelium, which allows for its use in primary prevention aimed at preventing death [60]. However, the described mechanisms also seem to be beneficial in secondary prevention. A randomized study conducted on 88 patients after myocardial infarction, in which participants received 500 mg of quercetin per day, showed that people from the studied group were characterized by a lower level of TNF-alpha (*p* = 0.009) and a higher level of total antioxidant capacity (for the control group 0.00 mmol/L and the studied group 0.024 mmol/L, respectively) [61]. In a study by Liu et al. conducted in a mouse model of myocardial infarction, it was proven that quercetin administration improved cardiac ejection fraction and reduced ventricular remodeling after infarction [62]. In a multicenter, pilot study by Kozhukhov et al., which aimed to investigate the cardioprotective effect of quercetin on reducing infarct size in patients with ST-segment elevation myocardial infarction (STEMI), the addition of quercetin to standard STEMI therapy was proven to reduce infarct size and prevent intramuscular hemorrhage after anterior STEMI. A significant limitation of this study was the small study population (n = 143). The infarct size assessed using creatine kinase-myocardial band area under curve (CK-MB AUC) was the primary study outcome. The study arms did not differ in demographics, time to admission, and main clinical data. The median early CK-MB AUC was significantly lower in the quercetin group than in the controls (8036 ± 7594 vs. 11,219 ± 8146 U × 1 h/L, *p* = 0.015). Intravenous quercetin administration was associated with less reperfusion-induced intramyocardial hemorrhage via Cardiac Magnetic Resonance on Day 3 (11.1% of patients in quercetin group vs. 53.3% of patients in control group, *p* < 0.024). There were no significant differences in the left ventricle ejection fraction and LV remodeling indicators [63].

Hypertension is one of the strongest cardiovascular risk factors, and WHO data show that almost 1.3 billion people worldwide are struggling with this disease, almost half of whom are unaware of the disease, and only one in five is effectively treated [64]. Hypertension is a disease that leads to a number of complications—renal, ophthalmological, and vascular. In recent years, we have not observed the emergence of new drugs registered for the treatment of hypertension. The pathophysiology of hypertension is very complex and has not been fully discovered. Many studies indicate the important role of the vascular endothelium and factors that disrupt its functioning [65]. Quercetin has shown many potentially beneficial effects on vascular endothelial function in many studies, which consequently led to a reduction in blood pressure. Increased reactive oxygen species contribute to the disruption of the vasodilatory action of nitric oxide by inactivating NO synthase [66]. The effect of quercetin on NO synthase is bidirectional, by reducing the level of reactive oxygen species and activating endothelial nitric oxide synthase (e-NOS) via AMPK, which consequently leads to vasodilation [67]. Other potential mechanisms include the activation of vasorelaxant Kca 1.1 channels with simultaneous inhibition of Ca channels [68]. A meta-analysis of five randomized studies showed that quercetin supplementation does not affect diastolic pressure, while its effect on systolic pressure is significant ((WMD): −1.9, 95% CI = −3.2 to −0.6, *I*^2^ = 88.3%) [69]. In the analysis conducted by Popiolek-Kalisz et al., which included 841 patients with normal or (pre)hypertensive blood pressure, it was proven that quercetin supplementation significantly decreased SBP in the mixed population (MD: −2.38 mmHg; 95% CI: −3.80–−0.96; *p* = 0.01) and in the normotensive subgroup (MD: −1.82 mmHg; 95% CI: −2.43–−1.20; *p* < 0.0001) and DBP in the (pre)hypertensive subgroup (MD: −3.14 mmHg; 95% CI: −4.44–−1.84; *p* < 0.00001). Quercetin supplementation decreases BP in normotensive and (pre)hypertensive patients [70].

The results from the study by Alugoju et al. show that quercetin in combination with calorie reduction significantly reduced hydrogen peroxide-mediated stress in yeast cells. They also showed that quercetin in combination with calorie reduction increased the percentage of yeast cell viability during chronological aging. The results of some studies suggest that quercetin may act as a modulator of cell signaling pathways related to carbohydrate metabolism and cell integrity, exerting a protective effect against oxidative stress [62,71,72].

## 5. Lipid Metabolism Disorders

Lipid metabolism regulation has been proved to be directly related to the development of atherosclerosis, which is one of the main causes of cardiovascular illnesses like ischemic heart disease and stroke [73,74]. The chronic and progressive pathology of this complex multifactorial disease is marked by endothelial dysfunction, low-grade inflammation in the walls of large- and medium-sized arteries, and lipid accumulation [75]. Various forms of lipoproteins and lipids are responsible for lipid trafficking, but classic LDL and HDL, cholesterol, oxidized LDL cholesterol, small dense LDL cholesterol, lipoprotein (a), and apolipoprotein B (ApoB) are those with clinical significance [76]. According to population studies, elevated levels of LDL cholesterol and ApoB, the main structural protein of LDL, have been linked to an increased risk of atherosclerotic cardiovascular events [77]. Oxidative changes in LDLs are associated with artery hardening. One of the most important steps in the oxidation hypothesis of atherogenesis is the modification of LDL to oxidized LDL (Ox-LDL). Oxygen-LDL turns into a potent chemoattractant that promotes the build-up of significant intracellular cholesterol deposits. Ox-LDL is then attracted by monocyte-derived macrophages, which are involved in the formation of foam cells constituting the atherosclerotic plaque’s core [78]. Statins, ezetimibe, or PCSK9 inhibitors are lipid-lowering therapies that have been shown in numerous clinical trials to dramatically improve cardiovascular outcomes [79]. Many clinical trials were previously conducted to determine the effects of regular consumption of quercetin on blood lipid levels. The double-blind randomized clinical study by Talirevic et al. showed diminution of the levels of cholesterol, TG, and LDL, and an increase in HDL levels [80]. Brull et al. investigated the effect of quercetin after regular administration on blood pressure (BP) in overweight or obese patients with prehypertension and stage I hypertension. In addition, potential mechanisms responsible for the hypothesized effect of quercetin on BP were examined. Subjects (n = 70) were randomly assigned to receive 162 mg/d quercetin from onion peel extract powder or a placebo in a double-blind, placebo-controlled, crossover study with 6-week treatment periods separated by a 6-week washout period. Ambulatory blood pressure (ABP) and office blood pressure were measured before and after the intervention, urine and blood samples were collected, and endothelial function was measured using EndoPAT technology. Quercetin significantly reduced daytime and nighttime systolic BP in subjects with hypertension, but had no significant effect in the between-group comparison. Supplementation with 162 mg/d of quercetin from onion peel extract reduced blood pressure in patients with hypertension, suggesting a cardioprotective effect of quercetin. The mechanisms responsible for the blood pressure reduction remain unclear [81]. Despite the fact that these two studies suggest quercetin may improve blood lipid levels, a meta-analysis of randomized controlled trials produced conflicting findings. Some meta-analyses demonstrated that quercetin, at doses greater than 50 mg/day, significantly reduces TG, but otherwise has no clinically significant effects on plasma lipids. Furthermore, it was discovered that the duration and dosage of quercetin supplementation had a significant impact on TG levels. As a result, while some trends can be seen, it is impossible to draw firm conclusions regarding the effectiveness of quercetin on hyperlipidemia due to the small number of clinical trials and the small sample size. The mechanism of quercetin’s antihyperlipidemic effect may involve multiple factors. By specifically blocking the absorption of intestinal cholesterol and lowering the expression of the epithelial cholesterol transporter Niemann-Pick C1-like 1 (NPC1L1), quercetin may be able to lower high blood cholesterol levels [82]. According to recent research, quercetin’s impact on inflammation and glycometabolism may be connected to its function in lipid metabolism. The study showed that using quercetin in in vitro tests and obese mouse models significantly reduced the expression levels of interleukin-1 (IL-1) and interleukin-6 (IL-6), which are inflammatory factors in adipocytes. Furthermore, the findings demonstrated that feeding obese mice quercetin led to a significant decrease in their body weight. Additionally, the expression of key adipose factors, such as CCAAT/enhancer binding protein (C/EBP), peroxisome proliferators-activated receptor γ (PPARγ), fatty acid-binding protein 4 (FABP4), and TG synthetases, was significantly downregulated [83]. The previous findings from transcriptome sequencing indicated that the adenosine monophosphate activated protein kinase (AMPK) was the primary signaling pathway for lipid metabolism [84]. The AMPK signaling pathway coordinates glucose metabolism by regulating glycolysis and gluconeogenesis, and it regulates lipid metabolism by acting on fatty acid synthesis and oxidation [85]. In the present investigation, quercetin supplementation significantly raised AMPK protein expression as well as the mRNA expression of AMPKγ, AMPKα1, AMPKα2, and AMPKβ2 [86]. The results suggest that AMPK activation was accompanied by increased PPARα and reduced PPARγ expression. Three distinct receptor subtypes, PPARα, PPARβ, and PPARγ, are known to be highly expressed in tissues with high capacities for lipid catabolism, including the liver, skeletal muscle, and brown adipose tissue. PPARs regulate fatty acid oxidation and disintegration, lipid transportation, and lipoprotein assembly by modulating the transcription of their downstream genes [87]. In this study, quercetin activated AMPK, which regulated PPAR expression, increased lipid β-oxidation, and consequently reduced fat deposition. Quercetin can activate the PPARγ signaling pathway as well as inhibit the activity of inflammatory factors induced by MAPK signaling pathway, which activates leptin signaling in adipose tissue and accelerates fat oxidation [88]. The effects of quercetin on blood lipids is presented in Table 2.

## 6. Limitations

The use of quercetin in medicine is limited for several reasons. There are no clear, large-scale clinical trials in humans. Most studies were conducted in vitro or on small populations of subjects. Different doses of quercetin were used at different time intervals, and the population size varied. Another factor related to the limited possibilities of using quercetin in clinical practice is its limited bioavailability. Due to its poor water solubility and the first-pass effect, the use of quercetin as a therapeutic molecule in clinical practice is severely limited [37]. There are studies that attempt to solve this problem by modifying the form of quercetin (e.g., in the form of nanoparticles), but this is also still not standard. Quercetin is a highly lipophilic molecule that can be combined with the structure of solid lipid nanoparticles (SLNs). The study by Varshosaz et al. showed that the IC50 (half-maximal inhibitory concentration) of quercetin in cholesterol SLN was about six times lower than that of free quercetin. Quercetin’s cellular penetration has been shown to be improved by sterol-containing solid lipid nanoparticles to target hepatocellular carcinoma cells [89]. Furthermore, quercetin-containing preparations differ in terms of the quality and concentration of the active substance. The lack of standardization and precise dosing guidelines hampers their use in medicine [90]. To date, there are relatively few studies on the potential interactions of quercetin with drugs. Lack of approval by relevant institutions: Many health authorities, such as the Food and Drug Administration (FDA) and European Medicines Agency (EMA), have not approved quercetin as a drug, but only as a dietary supplement. It is possible that in the future quercetin will be delivered to the diet in the form of soy protein isolate-citrus pectin composite hydrogels [91]. Poór et al. proved that quercetin metabolites are able to strongly displace warfarin from HSA, suggesting that high quercetin doses can strongly interfere with warfarin therapy [92].

This means that its use in the treatment of serious diseases requires further research and evidence of safety and efficacy.

## 7. Conclusions

Quercetin has a beneficial effect on the functioning of the vascular endothelium, mainly by improving its elasticity and reducing oxidative stress. These effects may contribute to improving the overall function of the circulatory system, reducing the risk of developing cardiovascular diseases. Regular consumption of quercetin may support the integrity of the endothelium, preventing its damage as a result of negative external factors, such as inflammation or excessive production of reactive oxygen species. Quercetin has strong anti-inflammatory properties. Studies indicate that it inhibits a number of inflammatory mediators, such as pro-inflammatory cytokines (IL-6, Il-17, TNF-α, and IL-1β) which may contribute to reducing chronic inflammation. Reducing inflammation is important in preventing the development of cardiovascular diseases, such as atherosclerosis, as well as in the treatment of existing inflammatory diseases. Quercetin has the potential to regulate the lipid profile, reducing the levels of total cholesterol and triglycerides while increasing the level of HDL cholesterol. Improving lipid metabolism may lead to a reduced risk of atherosclerosis and other cardiovascular diseases associated with dyslipidemia. Quercetin, through its effects on the vascular endothelium, inflammatory processes, and lipid metabolism, is a promising supplement for preventing and supporting the treatment of cardiovascular diseases. Quercetin, due to its anti-inflammatory, antioxidant, and lipid properties, is a valuable dietary component in the context of cardiovascular disease prevention. Its use may contribute to improving the health of the circulatory system and reducing the risk of diseases associated with endothelial dysfunction, inflammatory processes, and lipid disorders. Although the results of previous studies are promising, further clinical studies are necessary to fully determine the optimal doses of quercetin and the mechanisms of its action in the context of cardiovascular diseases.

## Figures and Tables

**Table 1:** Quercetin studies on vascular endothelium and inflammation.

Study Models	Cells/Organ	Quercetin Dosage	Results	Effects	Ref.
IN VITRO	Human alveolar epithelia A549 cells	At various concentrations (0–100 μM for 4 h before addition of LPS (0–50 μg/mL) for 6 h	No change with dose of 10, 20, and 50 μM	Decreases oxidative stress by preventing LPS-induced NOX2 expression and suppressing IκBα degradation and the nuclear translocation of NF-κB, resulting in a reduction in the levels of the inflammatory cytokines. Supports the antioxidant defense system, with effects on inflammation and oxidative stress (inhibits cytokines for AD via NF-kB and ERK1/2 MAPK pathways in HaCaT cells). May reverse the upregulation of ICAM-1, VCAM-1, and E-selectin via inhibiting the activation of NF-kB and AP-1—these mechanisms could all relieve the progress of atherosclerosis. Effect on inflammation, oxidative stress, apoptosis, and mitochondrial structure and function.	[34]
	Immortalized human HakaT keratinocyte	1.5 μM for 6 h	Supports the antioxidant defense system, with effects on inflammation and oxidative stress (inhibits cytokines for AD via NF-kB and ERK1/2 MAPK pathways).	Effect on inflammation, oxidative stress, apoptosis, and mitochondrial structure and function. Suppressive effects of quercetin on hydrogen peroxide-induced caveolin-1 phosphorylation in endothelial cells.	[10]
	vascular endothelial cells HUVECs	30 μg/mL for 18 h			[36]
	H9C2 cells immortalized myoblasts	50 μM after 2, 4, and 6 h exposition	Promotes the proliferation of H9C2 cells while inhibiting inflammation, oxidative stress, and cell apoptosis, and alleviates the structural and functional dysfunction of mitochondria—achieved by promoting PVT1 expression.	Quercetin and its metabolites improved vessel function by inducing eNOS activity via the phosphorylation of AMPK.	[40]
	HUVECs cells -endothelium	Treated with quercetin for 24 h and then exposed to hydrogen peroxide H_2_O_2_	Quercetin aglycone suppresses both H_2_O_2_-dependent Cav-1 phosphorylation and vascular permeability increases by reducing H2O2-induced VE-cadherin expression.	Quercetin treatment inhibited the increase in vascular permeability and reduced VE-cadherin expression.	[4]
	Human aortic endothelial cells	5 and 10 μM	Beneficial effects of quercetin on endothelial cell functions are in part mediated via AMPK pathway.		[55]
IN VIVO ANIMAL MODELS	C57BL/6 mice	30 mg/kg, oral administration		Effective in controlling *C. rodentium*-induced inflammation.	[3]
	Male Wistar rats	Orally 75 mg/kg for 14 days	Limits the cardiotoxic effect of cardiotoxic substances in the rat’s cardiac mitochondria—Q. Could be used as a protective agent.	Effects of quercetin against oxidative stress caused by cardiotoxic substances.	[52]
	Male Wistar rats at the age of 7–8 weeks	orally 30 mg/kg each day for 2 weeks	May have promising potential by modulating the AMPK/SIRT1/NF-κB signaling pathway.	Effects of quercetin on the AMPK and inflammatory/oxidative stress response in diabetic-induced atherosclerosis.	[39]
	Wistar–Kyoto (WKY) and SHR rats	10 mg/kg for 6 weeks	Promoted autophagy in endothelial cells under both normal and oxidative stress conditions. Quercetin protects endothelial function in metabolic disorders, and that autophagy is a potential target for intervention against vascular dysfunction.	Improves vascular endothelial function through the promotion of autophagy.	[49]
	Albino Wistar rats, male with diabetes	30 mg/kg and 60 mg/kg	Significantly attenuated the STZ-induced, diabetes-induced impairments in the behavioral, endothelial, and biochemical parameters.	Efficacy of quercetin in diabetes-induced vascular endothelium dysfunction and related dementia.	[32]
IN VIVO HUMANS	Patients with myocardial infarct	500 mg/day for 8 weeks	Associated with a decrease in multiple inflammatory cytokines, such as IL-1α, IL-1β, IL-2, IL-10, TNF-α, macrophage chemoattractant protein-1, and cyclooxygenase-2.	May significantly increase total antioxidant capacity and improve inflammatory factors and quality of life, but may not have a significant effect on inflammatory factors, blood pressure, and other dimensions of QOL.	[56]
	Endothelial dysfunction in age-related cardiovascular diseases	1000–1200 mg/day for 12 weeks	Decreased expression of adhesion molecules (ICAM-1, VCAM-1).	Further studies using longer periods of time and different doses of quercetin are warranted to establish this efficacy. There is no evidence of toxicity, but data on long-term safety are lacking. Some minor side effects such as mild headache, nausea, and tingling of the extremities. A possible mechanism is shown in increased cAMP levels and the inhibition of ADP-induced platelet aggregation.	[22]

**Table 2:** Effects of quercetin on blood lipids.

Authors	Subject of Study	Dose of Quercetin	Results
Edwards et al. (2007) [15]	Smokers with elevated risk of cardiovascular disease	500 mg/day for 4 weeks	Decrease in total cholesterol and LDL cholesterol No change in HDL cholesterol or triglycerides
Zhang, Y.F. et al. (2024) [16]	Healthy volunteers	150 mg/day for 6 weeks	Decrease in total cholesterol and LDL cholesterol No effect on HDL cholesterol or triglycerides
Egert et al. (2008) [17]	Healthy overweight and obese individuals	150 mg/day for 6 weeks	Decrease in serum total cholesterol and LDL cholesterol No effect on HDL cholesterol or triglycerides
Lee et al. (2011) [18]	Hypercholesterolemic individuals	100 mg/day for 10 weeks	Decrease in total cholesterol, LDL cholesterol, and triglycerides Increase in HDL cholesterol
Chen-yu, G. et al. (2012) [19]	Obese individuals	150 mg/day for 8 weeks	Decrease in LDL cholesterol and triglycerides No significant effect on total cholesterol or HDL cholesterol
Sahebkar (2017) [20]	Patients with metabolic syndrome	50 mg/day for 12 weeks	Decrease in total cholesterol, LDL cholesterol, and triglycerides Increase in HDL cholesterol

## Abbreviations

CV	Cardiovascular
CVS	cardiovascular system
CVDs	cardiovascular diseases
CAD	coronary artery disease
CHD	coronary heart disease
TNF-α	Tumor Necrosis Factor
Il-6: Il-1-β, Il-17, Il-8	Interleukin
NF-kB	Nuclear Factor kappa B
AMPK	5′AMP-activated protein kinase
AP-1	Activator Protein 1
SIRT1	Sirtuin 1
Nrf2	Nuclear factor erythroid 2-related factor 2
ICAM-1	Intercellular Adhesion Molecule 1
VCAM-1	Vascular cell adhesion protein 1
ROS	Reactive Oxygen Species
IgE	Immunoglobulin E
NLRP3	Family Pyrin Domain Containing 3
NADPH	Nicotinamide adenine dinucleotide phosphate
NOX2	Oxidase 2/cytochrome b(558)
MDA	Malondialdehyde
4-HNE	4-Hydroxynonenal
Cd39	Ectonucleoside triphosphate diphosphohydrolase-1 (gene: ENTPD1)
*p*-Cav-1	Phospho-Caveolin-1 (Tyr14) Antibody
Eph/EphB4	Ephrin/Ephrin type-B receptor 4
eNOS/iNOS	Endothelial Nitric Oxide Synthase/Inducible nitric oxide synthase
Ca2	Carbonic anhydrase II
NO	Nitric Oxide
TG	Triglycerides
